# NUSAP1 potentiates chemoresistance in glioblastoma through its SAP domain to stabilize ATR

**DOI:** 10.1038/s41392-020-0137-7

**Published:** 2020-04-22

**Authors:** Yuzu Zhao, Jiang He, Yongsen Li, Shengqing Lv, Hongjuan Cui

**Affiliations:** 1 State Key Laboratory of Silkworm Genome Biology, Southwest University, Chongqing, China; 2 Cancer Center, Medical Research Institute, Southwest University, Chongqing, China; 3 Department of Neurosurgery, Xinqiao Hospital, Third Military Medical University, Chongqing, China

**Keywords:** CNS cancer, CNS cancer, RNAi, Predictive markers, Prognostic markers

## Abstract

NUSAP1, which is a microtubule-associated protein involved in mitosis, plays essential roles in diverse biological processes, especially in cancer biology. In this study, NUSAP1 was found to be overexpressed in GBM tissues in a grade-dependent manner compared with normal brain tissues. NUSAP1 was also highly expressed in GBM patients, dead patients, and GBM cells. In addition, NUSAP1 was found to participate in cell proliferation, apoptosis, and DNA damage in GBM cells. Ataxia telangiectasia and Rad3-related protein (ATR) are a primary sensor of DNA damage, and ATR is also a promising target in cancer therapy. Here, we found that NUSAP1 positively regulated the expression of ATR. Mechanistically, NUSAP1 suppressed the ubiquitin-dependent proteolysis of ATR. The SAP (SAF-A/B, Acinus, and PIAS) domain is a common motif of many SUMO (small ubiquitin-like modifier) E3 ligases, and this domain is involved in substrate recognition and ligase activity. This study further demonstrated that the SAP domain of NUSAP1 promoted the sumoylation of ATR, and thereby antagonized the ubiquitination of ATR. These results suggest that NUSAP1 stabilizes ATR by sumoylation. Moreover, NUSAP1 potentiated chemotherapeutic resistance to temozolomide (TMZ) and doxorubicin (DOX) through its SAP domain. Overall, this study indicates that NUSAP1 is a promising therapeutic target in GBM.

## Introduction

Glioblastoma multiforme (GBM), also called grade IV astrocytoma, along with other gliomas, constitutes the vast majority of malignant brain tumors.^1,2^ GBM is the most aggressive brain tumor with high mortality, poor prognosis, and short survival.^3^ It is urgent to explore more therapeutic options for GBM diagnosis and treatment to further prolong the survival of GBM patients.

DNA damage is a process induced by various factors, including chemical carcinogens,^4^ radiation,^5^ and genomic agents.^6^ Once DNA damage occurs, cells initiate the DNA damage response (DDR). Consequently, genes involved in DNA damage processing are targets for tumor therapy.^7–10^ Ataxia telangiectasia mutated (ATM) together with ataxia telangiectasia and Rad3-related protein (ATR) and DNA-dependent protein kinase catalytic subunit (DNA-PK_CS_) constitutes the core of the DDR.^11^ DNA damage agents have shown favorable efficacy in anticancer therapy, which has accelerated the development of cancer chemotherapy. However, in addition to the toxicity of these agents, chemotherapeutic resistance occurs frequently, which directly hinders cancer therapy.^12–14^ Chemotherapeutic resistance is one of the reasons for the poor prognosis of GBM. There is still an urgent need to find more targets to enhance the effectiveness of chemotherapy in GBM.

Nucleolar and spindle-associated protein 1 (NUSAP1) is a microtubule-associated protein (MAP) participating in mitotic spindle organization.^15^ In addition to the essential role of NUSAP1 in mitosis, NUSAP1 is involved in many other biological processes as follows. NUSAP1 plays an essential role in chromosomal segregation and kinetochore microtubule dynamics.^16,17^ NUSAP1 is an SCF^cyclin F^ substrate that participates in ubiquitin-dependent proteolysis.^18^ Moreover, NUSAP1 serves as an important regulator in cancer biology. NUSAP1 regulates metastasis of cervical carcinoma by Wnt/β-catenin signaling.^19^ NUSAP1 promotes aggressiveness in astrocytoma through Hedgehog signaling.^20^ NUSAP1 is also regulated by other factors, such as E2F1,^21^ c-Myc,^22^ and miR193a-5p.^23^ In addition, NUSAP1 acts as an immunogenic antigen in most AML patients, which implies that NUSAP1 is a tumor antigen.^24^ NUSAP1 promotes migration and invasion in renal cell carcinoma, as well as colorectal cancer.^25,26^ NUSAP1 is regarded as a candidate biomarker in oral squamous cell carcinoma,^27^ esophageal squamous cell carcinoma, ^28^ cervical cancer,^19^ breast cancer,^29^ hepatocellular carcinoma,^23^ and prostate cancer.^21^ NUSAP1 is also highly expressed in astrocytoma.^20,30,31^ Despite the extensive study of NUSAP1, whether NUSAP1 participates in DNA damage as well as chemotherapeutic resistance in GBM remains unclear; herein, the role of NUSAP1 in GBM was investigated. This study indicates that NUSAP1 acts as a tumor-related gene in GBM, and that NUSAP1 inhibits cell proliferation and induces apoptosis and DNA damage in GBM. Moreover, NUSAP1 contributes to GBM chemotherapeutic resistance. This study indicates that NUSAP1 serves as a candidate indicator in GBM diagnosis as well as in GBM therapy.

## Results

### NUSAP1 is highly expressed in GBM patients as well as in GBM cells

**Fig. 1:**
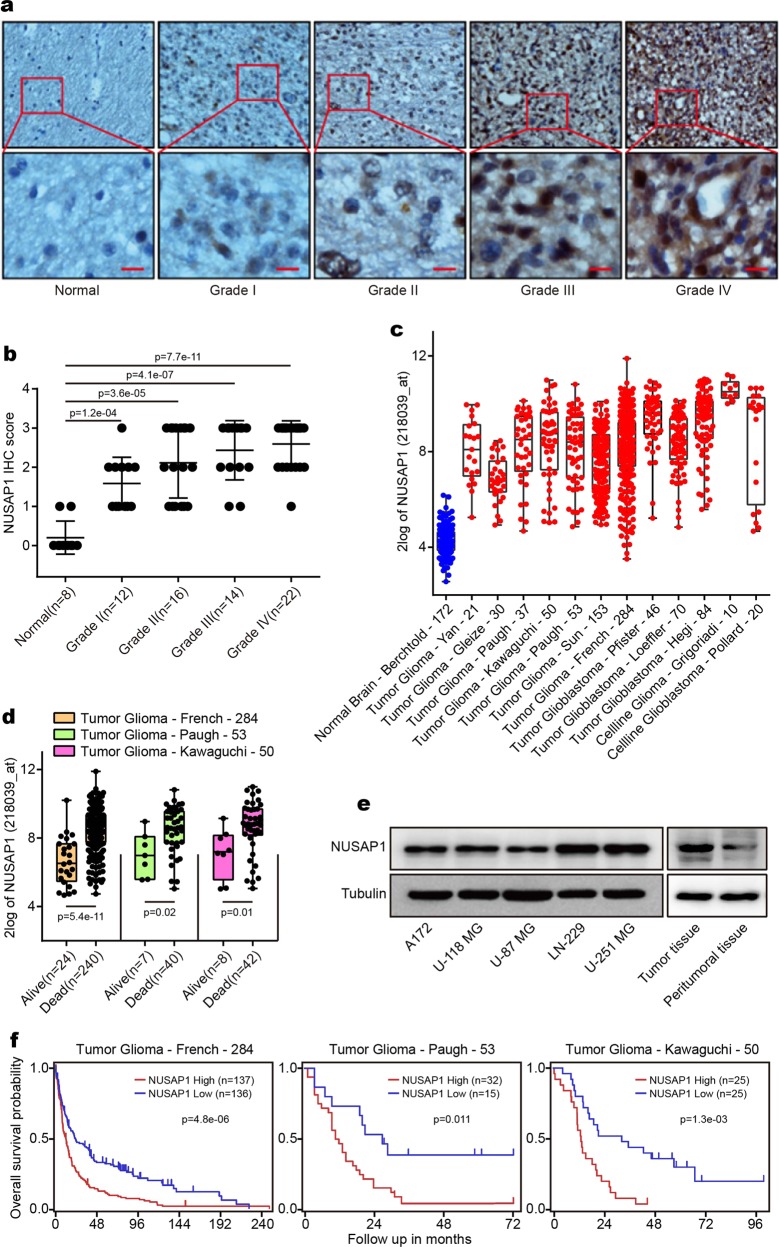
NUSAP1 is highly expressed in GBM patients and in GBM cells. **a** Immunohistochemical analyses of NUSAP1 in normal brains and glioma tissues. **b** Statistical analyses of NUSAP1 in 8 normal brains and 64 glioma tissues. **c** The expression of NUSAP1 in normal brain as well as in glioma patients and in cell lines from 13 different data sets. **d** Analyses of NUSAP1 in living patients and in dead patients from three different databases. **e** The level of NUSAP1 in five GBM cell lines as well as in a pair of peritumoral and tumor tissues. **f** Kaplan–Meier analysis of progression-free survival using data from three different glioma databases

To investigate the role of NUSAP1 in GBM, an immunochemistry (IHC) assay was performed to detect the expression of NUSAP1 in 72 normal brain and glioma samples. NUSAP1 was highly expressed in glioma and GBM samples in a grade-dependent manner (Fig. 1a, b). To further determine the role of NUSAP1 in the prognosis of GBM patients, the prognostic value of NUSAP1 was analyzed in the R2: Genomics Analysis and Visualization Platform. In comparison with that in the normal brain control, the level of NUSAP1 was upregulated in 12 glioma and GBM data sets, not only in patients but also in cell lines (Fig. 1c). The expression of NUSAP1 was higher in dead patients than in living patients according to three different data sets (Fig. 1d). Furthermore, a high level of NUSAP1 predicted poor prognosis in four different data sets (Supplementary Fig. S1a). NUSAP1 was expressed in a grade-dependent manner in glioma patients (Supplementary Fig. S1b, c). The level of NUSAP1 in several GBM cell lines was detected. The results showed that NUSAP1 was highly expressed in all of these cell lines. We further examined NUSAP1 in a pair of peritumoral and tumor tissues, and NUSAP1 was upregulated in the tumor tissue (Fig. 1e). In addition, high expression of NUSAP1 indicated poor prognosis in three glioma data sets (Fig. 1f).

### Downregulation of NUSAP1 inhibits cell proliferation and induces apoptosis in GBM cells

**Fig. 2:**
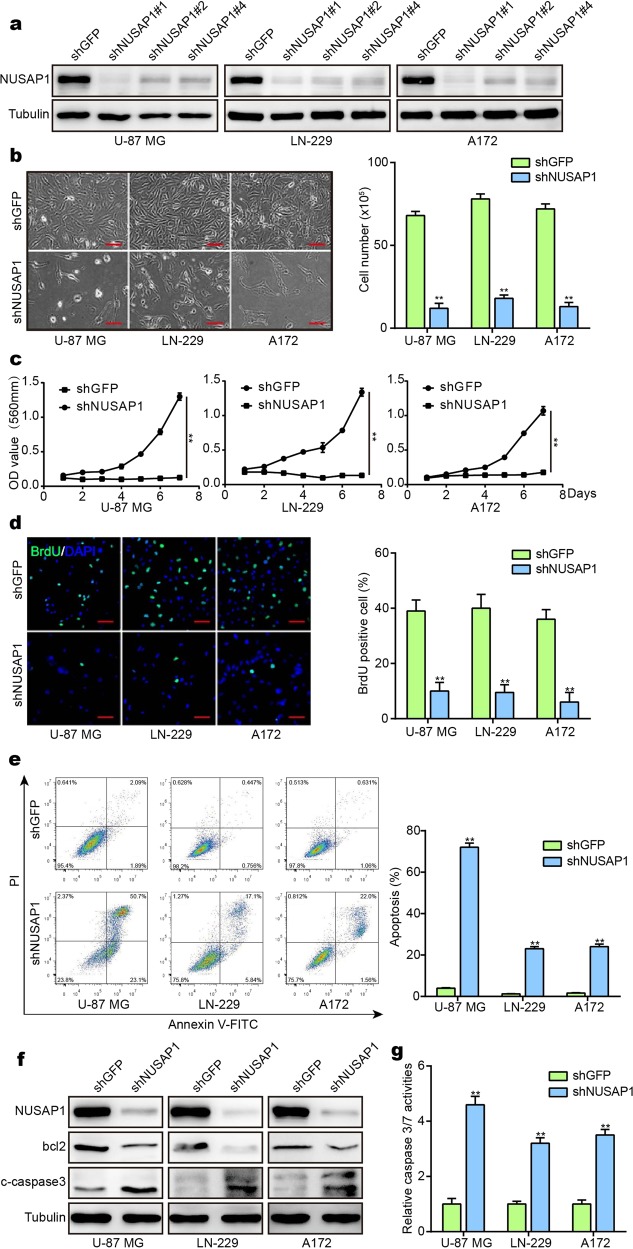
Silencing NUSAP1 inhibits cell proliferation and causes apoptosis in GBM cells. **a** Western blot analyses of NUSAP1 in cells with NUSAP1 knockdown. **b** The morphology and cell number of GBM cells after knocking down NUSAP1. **c** Viability of NUSAP1-knockdown GBM cells. **d** BrdU-positive GBM cells after knocking down NUSAP1. **e** Flow cytometry analyses of apoptosis in GBM cells with NUSAP1 knockdown. **f** The expression of the apoptotic proteins bcl2 and cleaved caspase-3 in cells with NUSAP1 knockdown. **g** Caspase-3/7 activity of GBM cells with NUSAP1 knockdown. All data are expressed as the mean ± SD, and significant differences were determined by Student’s *t* test. *P < 0.05, **P < 0.01, ***P < 0.001. *P* < 0.05 was considered statistically significant

A high level of NUSAP1 in GBM indicates that NUSAP1 acts as an indicator for the diagnosis and prognosis of GBM. These results suggest that NUSAP1 can serve as a tumor promoter in GBM. To test this hypothesis, three independent short hairpin RNAs (shRNAs) were designed to knock down NUSAP1 in the GBM cell lines U-87 MG, LN-229, and A172. Western blot analysis showed that NUSAP1 was successfully knocked down by the three shRNAs (Fig. 2a). Cell viability and DNA synthesis were detected, and the results showed that cell viability and DNA synthesis were inhibited by these three shRNAs (Supplementary Fig. S2a, b). ShNUSAP1#1 was then used to downregulate the expression of NUSAP1 in subsequent experiments, since shNUSAP1#1 presented the highest efficiency. NUSAP1 plays an essential role in mitosis, and cell proliferation after knocking down NUSAP1 was assessed. By microscopy, GBM cells with NUSAP1 knockdown showed significant morphological changes, and the cell numbers sharply decreased (Fig. 2b). Cells with NUSAP1 knockdown showed a decline in the growth curve (Fig. 2c). DNA synthesis was also reduced in NUSAP1-knockdown cells (Fig. 2d). After knocking down NUSAP1 in GBM cell lines, cells died significantly faster than control cells. We therefore examined apoptosis by flow cytometry. The proportion of apoptotic cells increased in NUSAP1-knockdown cells (Fig. 2e). To further validate the results above, western blotting was performed. The levels of the apoptosis-related protein bcl2 and the apoptotic marker cleaved caspase-3 were distinctly altered, with bcl2 decreasing and cleaved caspase-3 increasing upon NUSAP1 depletion (Fig. 2f). In addition, caspase-3/7 activity was detected in cells from the control group and NUSAP1-knockdown group. Consistent with the results obtained above, caspase-3/7 activity increased sharply after knocking down NUSAP1 (Fig. 2g).

### NUSAP1 knockdown causes DNA damage in GBM cells

**Fig. 3:**
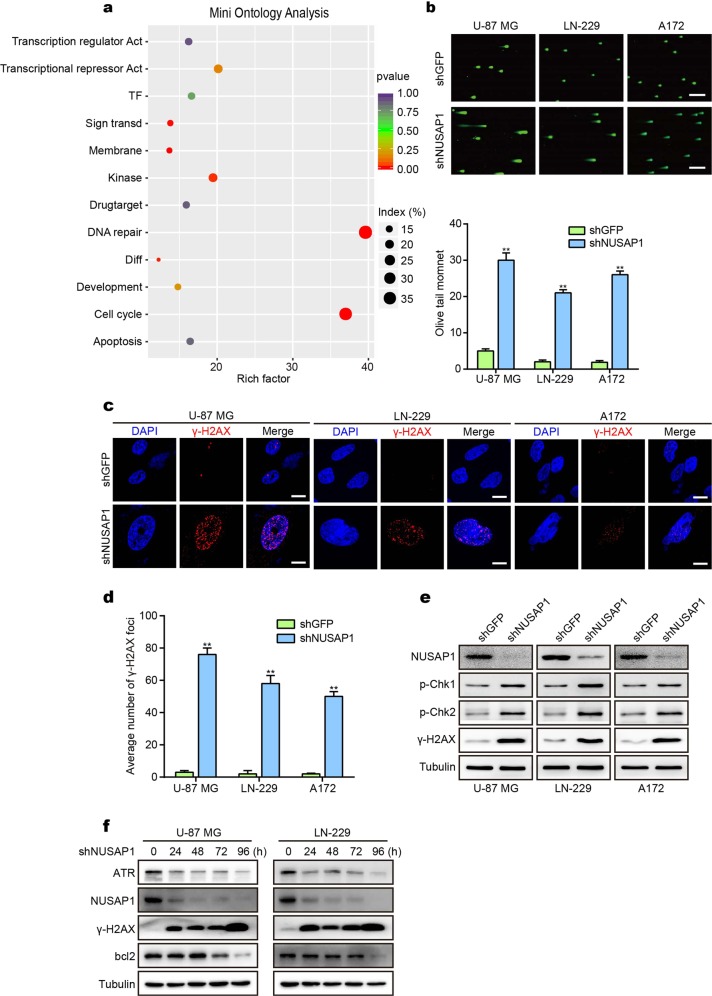
Depletion of NUSAP1 induces DNA damage in GBM cells. **a** Mini ontology obtained in French’s database using gene sets related to NUSAP1, which were identified by a significant difference of *P* < 0.001. **b** Tailed DNA in single cells depleted of NUSAP1. **c** IF staining of γ-H2AX in NUSAP1-knockdown GBM cells. **d** Quantification of γ-H2AX foci in the indicated GBM cells. **e** Western blot analyses of DDR proteins in GBM with NUSAP1 knockdown. **f** The level of the indicated proteins in GBM cells with NUSAP1 knockdown for 0 h, 24 h, 48 h, 72 h, and 96 h. All data are used as the mean ± SD, and significant differences were determined by Student’s *t* test. *P < 0.05, **P < 0.01, ***P < 0.001. *P* < 0.05 was considered statistically significant

We constructed a mini ontology in French’s database using gene sets related to NUSAP1, which were identified by a significant difference of *P* < 0.001. We found that NUSAP1 was significantly correlated with the DNA repair process (Fig. 3a). We hypothesized that NUSAP1 acted as a regulator in the DDR to dictate cell fate. To confirm this hypothesis, a comet assay was performed to assess DNA damage through single-cell gel electrophoresis. A large number of cells with tailed DNA were observed after knocking down NUSAP1. In contrast, cells with tailed DNA were rare in the control group (Fig. 3b). Phosphorylated histone H2AX (ɣH2AX) is an indicator of the DDR. ɣH2AX is recruited to lesions when DNA damage occurs.^32^ An immunofluorescence (IF) assay was performed to examine ɣH2AX levels. Positive cell staining for ɣH2AX increased in NUSAP1-knockdown cells compared with cells in the control group (Fig. 3c, d). To further validate the results acquired above, the active forms of proteins involved in the DDR were detected, and the results showed that P-CHK1, P-CHK2, and ɣH2AX levels were upregulated in the NUSAP1-knockdown group (Fig. 3e). Overall, knocking down NUSAP1 induces apoptosis and DNA damage. We then detected bcl2 and ɣH2AX at different time points, and the results showed that DNA damage occurs at 24 h after knocking down NUSAP1, while apoptosis occurs at 72 h, which reminds us that DNA damage induced by NUSAP1 occurs earlier than apoptosis (Fig. 3f).

### NUSAP1 stabilizes ATR through its SAP domain to promote sumoylation of ATR

**Fig. 4:**
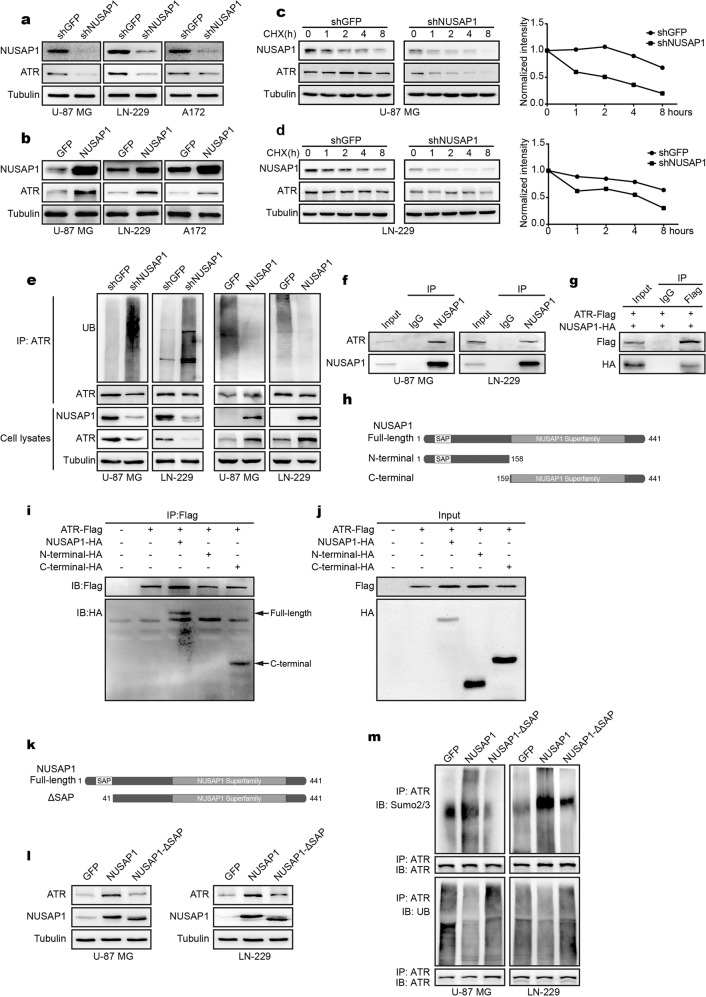
NUSAP1 stabilizes ATR by sumoylation. **a** The level of ATR in cells with NUSAP1 knockdown. **b** The level of ATR in cells overexpressing NUSAP1. **c** The level of the indicated proteins in NUSAP1-knockdown U-87 MG cells treated with 100 µg/ml CHX for 0 h, 1 h, 2 h, 4 h, or 8 h. **d** The levels of the indicated proteins in NUSAP1-knockdown LN-229 cells treated with 100 µg/ml CHX for 0 h, 1 h, 2 h, 4 h, or 8 h. **e** The level of ubiquitinated ATR in the indicated GBM cells. **f** Interaction of endogenous NUSAP1 with endogenous ATR. **g** Interaction of exogenous NUSAP1 with exogenous ATR. **h** Diagram of different domains of NUSAP1. **i**, **j** Interaction of ATR with truncated NUSAP1 in 293FT cells. **k** Diagram of the SAP domain on the N-terminus of NUSAP1. **l** The level of ATR in NUSAP1-overexpressing cells and in NUSAP1-ΔSAP-overexpressing cells. **m** The expression of sumoylated ATR and ubiquitinated ATR in NUSAP1-overexpressing cells and in NUSAP1-ΔSAP-overexpressing cells, respectively

During the detection of DDR-related proteins, we found that ATR levels decreased significantly in NUSAP1-knockdown cells (Fig. 4a). To further investigate whether NUSAP1 regulates ATR in cancer biology, NUSAP1 was stably overexpressed in the U-87 MG, LN-229, and A172 cell lines. ATR levels increased after NUSAP1 overexpression (Fig. 4b). Interestingly, knocking down NUSAP1 did not affect the mRNA level of ATR. NUSAP1 overexpression did not affect the mRNA level of ATR (Supplementary Fig. S3a). We assume that NUSAP1 regulates ATR through posttranslational modification (PTM). The effects of NUSAP1 depletion on ATR levels were measured by CHX chase assay. As expected, knocking down NUSAP1 significantly decreased the stability of ATR in comparison with the control group (Fig. 4c, d). In contrast, NUSAP1 overexpression stabilizes ATR (Supplementary Fig. S3b). Moreover, we performed a ubiquitin assay to detect the ubiquitination level of ATR. The results indicated that the ubiquitination level of ATR was increased after NUSAP1 depletion, while the ubiquitination level of ATR was decreased when NUSAP1 was overexpressed (Fig. 4e). To further investigate the role of NUSAP1 in stabilizing the ATR protein, we detected the interaction of NUSAP1 and ATR. An IP assay was performed. As expected, both endogenous and exogenous NUSAP1 interacted with ATR (Fig. 4f, g). Furthermore, truncated forms of NUSAP1 were cotransfected with full-length ATR. The results indicated that the C-terminus of NUSAP1 interacted with ATR (Fig. 4h–j). Previous studies have demonstrated that NUSAP1 contains an SAP domain.^33^ SAP domains are commonly found in many SUMO E3 ligases. SAP domains have also been implicated in substrate recognition and ligase activity. Sumoylation has been indicated to antagonize ubiquitin-dependent degradation.^34,35^ To detect whether the SAP domain of NUSAP1 regulates sumoylation to antagonize the ubiquitination of ATR, NUSAP1-ΔSAP (amino acids 41–441) with deletion of its SAP domain was constructed. Not surprisingly, NUSAP1-ΔSAP overexpression decreased ATR levels compared with the NUSAP1 overexpression group (Fig. 4k, l). Furthermore, NUSAP1-ΔSAP overexpression reversed the sumoylation and ubiquitination of ATR induced by NUSAP1 overexpression (Fig. 4m). In addition, sumoylation of ATR was assessed after knocking down NUSAP1. As expected, downregulation of NUSAP1 reduced the sumoylation level of ATR (Supplementary Fig. S3c).

### NUSAP1 promotes chemotherapeutic resistance through its SAP domain

**Fig. 5:**
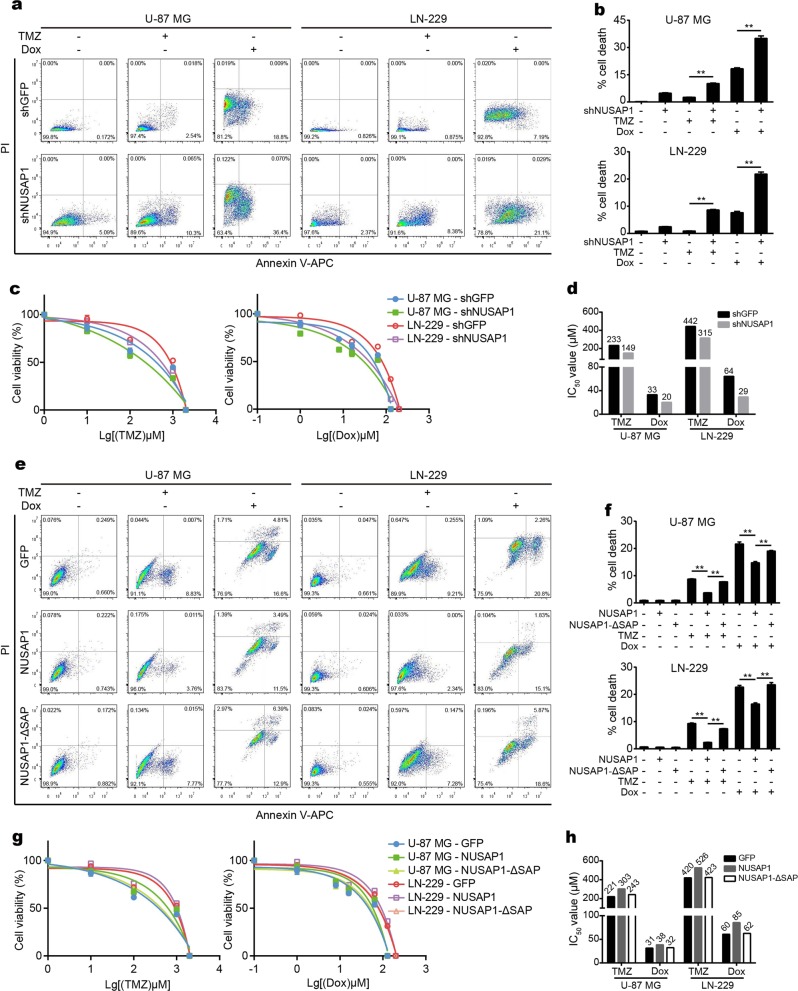
NUSAP1 potentiates chemoresistance in GBM cells. **a** Flow cytometry analyses of apoptosis in cells with NUSAP1 knockdown in response to the chemotherapeutic agents TMZ and DOX. **b** Quantification of the apoptotic rate in (**a**). **c** Viability of GBM cells depleted of NUSAP1 in response to TMZ or DOX. **d** IC_50_ value of the indicated cells treated with TMZ or DOX. **e** Flow cytometry analyses of apoptosis in NUSAP1-overexpressing cells and in NUSAP1-ΔSAP-overexpressing cells. **f** Quantification of the apoptotic rate in (**e**). **g** Viability of NUSAP1- and NUSAP1-ΔSAP-overexpressing GBM cells treated with TMZ or DOX. **h** IC_50_ value of the indicated cells treated with TMZ or DOX. All data are expressed as the mean ± SD, and significant differences were determined by Student’s *t* test. *P < 0.05, **P < 0.01, ***P < 0.001. *P* < 0.05 was considered statistically significant

NUSAP1 regulates the stability of ATR in GBM cells according to the results mentioned above. ATR acts as an essential factor in DNA damage as well as the DDR, and plays important roles in chemotherapeutic resistance.^36^ To validate whether NUSAP1 is involved in the regulation of chemotherapeutic sensitivity, we treated GBM cells with two chemotherapeutic agents, TMZ and DOX. After knocking down NUSAP1, cells treated with both TMZ and DOX showed higher apoptosis (Fig. 5a, b). In addition, both TMZ and DOX treatment reduced the viability of NUSAP1-downregulated cells (Fig. 5c). NUSAP1 depletion resulted in a lower IC_50_ toward both TMZ and DOX than the control group (Fig. 5d). Furthermore, caspase-3/7 activity was detected. Cells treated with TMZ and DOX after knocking down NUSAP1 showed higher caspase-3/7 activity (Supplementary Fig. S4a). In contrast, cells with NUSAP1 overexpression had lower apoptosis after treatment with these two agents. As expected, deletion of the SAP domain reversed this effect (Fig. 5e, f). Overexpression of NUSAP1 enhanced the viability of cells treated with TMZ and DOX. Deletion of the SAP domain restored this effect (Fig. 5g). NUSAP1 overexpression showed a higher IC_50_ toward TMZ and DOX, while deletion of the SAP domain showed a lower IC_50_ than the NUSAP1-overexpressing group (Fig. 5h). In addition, NUSAP1-overexpressing cells treated with TMZ and DOX presented lower caspase-3/7 activity, while deletion of SAP resulted in higher caspase-3/7 activity than the NUSAP1-overexpressing group (Supplementary Fig. S4b). To investigate whether NUSAP1 plays the roles mentioned above by stabilizing ATR, ATR was overexpressed after knocking down NUSAP1, and the results showed that ATR rescued ɣH2AX and cleaved-caspase9 in cells with NUSAP1 knockdown (Supplementary Fig. S5a). In addition, overexpression of ATR rescued apoptosis in cells with NUSAP1 knockdown upon treatment with TMZ (Supplementary Fig. S5b). These results remind us that NUSAP1 is involved in apoptosis, DNA damage, and chemoresistance by stabilizing ATR.

### Downregulation of NUSAP1 in GBM inhibits colony formation in vitro and tumorigenesis in vivo

**Fig. 6:**
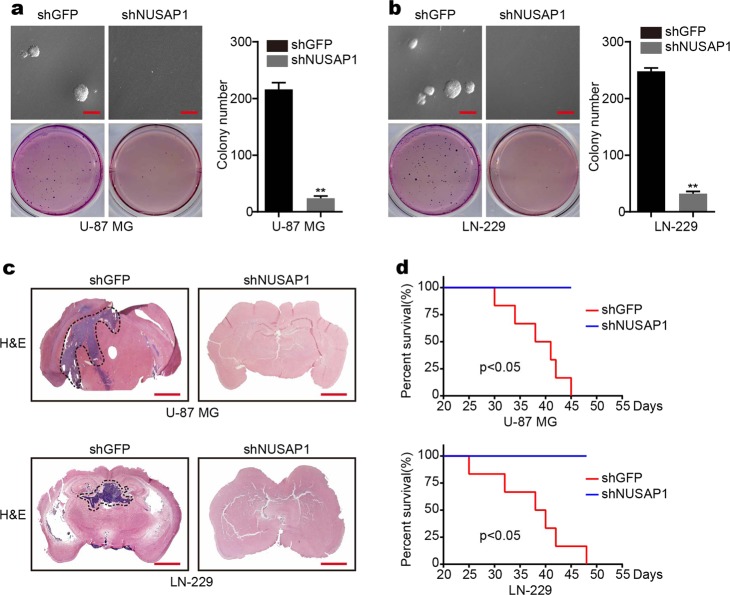
Silencing of NUSAP1 inhibits colony formation in vitro and tumorigenesis in vivo. **a** The colony-formation ability of U-87 MG cells with NUSAP1 knockdown. **b** Colony-formation ability of LN-229 cells with NUSAP1 knockdown. **c** Orthotopic tumorigenesis abilities of GBM cells depleted of NUSAP1. **d** Survival rates of mice with NUSAP1 knockdown

To further validate whether NUSAP1 affects clonogenicity in GBM cells, a soft agar assay was performed, and the results demonstrated that downregulation of NUSAP1 inhibited colony-formation ability (Fig. 6a, b). In addition, the effects of NUSAP1 on tumorigenesis were measured in an orthotopic implantation model. The results showed that NUSAP1 depletion significantly inhibited tumor formation in vivo. In fact, there were no tumors in the NUSAP1 depletion group (Fig. 6c). Furthermore, NUSAP1 depletion clearly prolonged the survival of the mice compared with the control group (Fig. 6d).

## Discussion

This study indicates that NUSAP1 acts as a tumor promoter in GBM. To summarize, NUSAP1 was overexpressed in glioma patients in a grade-dependent manner compared with normal brains. According to these results, NUSAP1 expression increased significantly even in grade I glioma (Fig. 1a, b). This indicates that NUSAP1 may be utilized as a diagnostic marker of glioma and GBM at an early stage. In addition, the level of NUSAP1 in each grade showed a significant difference (Fig. 1a, b; Supplementary Fig. S1b, c). This finding is significant, since NUSAP1 may act as a marker of grade-dependent diagnosis by quantifying the level of NUSAP1. Subsequent validation and utilization of this clinical significance requires large-scale analysis of NUSAP1 levels in patients at each grade of glioma. Furthermore, the level of NUSAP1 was enhanced in dead patients who had been diagnosed with glioma (Fig. 1d), and patients with high expression of NUSAP1 showed lower survival (Supplementary Fig. S1a). These results suggest that NUSAP1 may serve as an indicator of GBM prognosis.

ATR is considered a promising target in cancer therapy.^8,37,38^ This study showed that NUSAP1 contributed to cell proliferation, apoptosis, and DNA damage in GBM cells (Figs. 2, 3). In addition, knocking down NUSAP1 inhibited the expression of ATR, while overexpression of NUSAP1 enhanced the level of ATR in GBM cells (Fig. 4a, b). These findings indicate that NUSAP1 positively regulates the level of ATR. We speculated that inhibition of NUSAP1 would hinder DDR processes through depletion of ATR. DDR failure inhibits proliferation and induces apoptosis in GBM cells. Consequently, NUSAP1 depletion suppressed tumor progression in GBM. Therefore, NUSAP1 serves as a promising therapeutic target in GBM. Specific inhibitors of NUSAP1 can be evaluated and used in GBM therapy.

Ubiquitination and sumoylation are processes that function by covalently attaching to the lysine of the indicated substrates and then modifying the proteins through posttranslational modification (PTM). Ubiquitination often targets the indicated substrates for degradation through a proteasome-dependent pathway.^39,40^ Sumoylation does not promote protein degradation. In contrast, proteins modified by sumoylation have increased stability, which means that sumoylation stabilizes proteins.^34,35^ PIAS proteins (protein inhibitors of activated STAT) are the most common family of SUMO E3 ligases,^41^ and the N-termini of these proteins share a conserved SAP domain. The SAP domain of these proteins has substrate recognition and ligase activity.^42^ Previous studies have indicated that NUSAP1 contains an obvious SAP domain at its N-terminus.^15,33^ This study demonstrated that ATR bound to the C-terminus of NUSAP1 (Fig. 4h–j). The SAP domain at the N-terminus of NUSAP1 promoted sumoylation of ATR. Sumoylation of ATR then antagonized the ubiquitination of ATR (Fig. 4k–m). Ultimately, the SAP domain of NUSAP1 stabilized ATR.

This study showed that NUSAP1 positively regulated the level of ATR. A previous study indicated that ATR contributes to the chemotherapeutic resistance of GBM and malignant melanoma.^36^ Whether NUSAP1 is involved in the chemotherapeutic resistance of GBM remains unclear. Here, we addressed the role of NUSAP1 in the chemotherapeutic resistance of GBM. As expected, knocking down NUSAP1 increased the sensitivity of GBM cells to TMZ and DOX treatment. In contrast, overexpression of NUSAP1 increased the resistance of GBM cells to TMZ and DOX treatment. Moreover, deletion of the SAP domain of NUSAP1 increased chemotherapeutic sensitivity (Fig. 5). These results indicate that knocking down NUSAP1 enhances the vulnerability to chemotherapy in GBM cells. In summary, this study indicates that NUSAP1 serves as a promising target in GBM therapy. Moreover, since NUSAP1 is involved in chemoresistance, a promising therapeutic strategy that combines NUSAP1 inhibition with chemotherapy should be constructed.

## Materials and methods

### Cell lines and cell culture

All human GBM cell lines, including A172, U-118 MG, U-87 MG, LN-229, and U-251 MG, and the human embryonic kidney cell line 293FT were obtained from American Type Culture Collection (ATCC, Beijing, China). These cell lines were mycoplasma negative. All cells were cultured as described previously.^43^

### Reagents and antibodies

Antibodies against NUSAP1 (#12024-1-AP) and SUMO2/3 (#11251-1-AP) were purchased from the Proteintech Group (Wuhan, China). The NUSAP1 antibody (#H00051203-B01P) was purchased from Novus Biologicals (Colorado, USA). Anti-cleaved-caspase-3 (#9664), anti-phospho-chk1 (#2348), anti-phospho-chk2 (#2197), anti-ubiquitin (#3936), and anti-HA (#3724) antibodies were obtained from Cell Signaling Technology (Shanghai, China). Antibodies against HA (#AH158) and Flag (#AF519) were purchased from Beyotime (Shanghai, China). Anti-ATR antibody (#A300-138A) and anti-gamma-H2AX antibody (#A300-081A) were purchased from Bethyl (Texas, USA). Anti-tubulin antibody (#A5032) was purchased from Bimake (Shanghai, China). Anti-bcl2 antibody (#DB132) was obtained from DB Biotech (Kosice, Slovakia). MG132 (#S2619), TMZ (#S1237), and DOX (#S1208) were obtained from Selleck Chemicals (Shanghai, China). Cycloheximide, (CHX, #C7698), sodium deoxycholate (#D6750), N-ethylmaleimide (#E3876), and bromophenol blue (#114391) were purchased from Sigma-Aldrich (Shanghai, China). A rabbit enhanced polymer detection system (#PV-9001) for immunohistochemistry (IHC) was purchased from ZSGB-Bio (Beijing, China). ECL reagents were obtained from Beyotime (#P0018, Shanghai, China) and Clinx (#1810212, Shanghai, China). A comet assay kit was purchased from Trevigen group (#4250-050-K, Maryland, USA). Alexa Fluor 594-labeled secondary antibody (#A11034) and Hoechst 33342 (#H1399) were purchased from Thermo Fisher (Shanghai, China).

### IHC staining

IHC staining in 72 clinical glioma samples was performed following the manufacturer’s instructions. Briefly, tissues embedded in paraffin were deparaffinized following hydration and antigen retrieval, and then incubated with anti-NUSAP1 antibody (1:100). Tissues were then visualized with a rabbit enhanced polymer detection system, and hematoxylin was used for counterstaining. Images were obtained by microscopy.

### Patient data analysis

The expression of NUSAP1 in patients was analyzed using the R2: Genomics Analysis and Visualization Platform database as described previously.^44^

### Western blot analysis

Western blotting was performed as described previously.^43^

### Plasmids, transfection, and infection

NUSAP1-specific short hairpin RNAs (shRNAs) were purchased from Sigma-Aldrich and then cloned into the PLKO.1 vector. The sequences are as follows: shNUSAP1#1: 5′-CCGGCCTCAGGTAACAGAGATTCAACTCGAGTTGAATCTCTGTTACCTGAGGTTTTTTG-3′; shNUSAP1#2: 5′-CCGGGAGCACCAAGAAGCTGAGAATCTCGAGATTCTCAGCTTCT TGGTGCTCTTTTTTG-3′; and shNUSAP1#4: 5′-CCGGGAACCACACAAAGGAAAGCTACTCGAGTAGCTTTCCTTTGTGTGGTTCTTTTTTG-3′. The sequences encoding human NUSAP1 included the full length of NUSAP1 (amino acids 1–441), the N-terminus of NUSAP1 (amino acids 1–158), the C-terminus of NUSAP1 (amino acids 159–441), and NUSAP1-ΔSAP (amino acids 41–441), which were cloned into the pCDH-CMV-MCS-EF1-GFP-Puro vector and were provided by GeneCreate. For transient transfection, plasmids encoding full-length ATR and truncated NUSAP1 were collectively transfected into 293FT cells by Lipofectamine 2000. Stable transfection was performed as described previously.^43^

### Flow cytometry analysis

Flow cytometry analysis was performed as described previously.^44^

### Comet assay

A comet assay to detect DNA damage in cells was performed following the manufacturer’s instructions. Briefly, cells were harvested and counted to 1 × 10^5^/ml, and mixed with molten LM agarose at a ratio of 1:10 (V/V) and immediately pipetted into 50 µl of mixture onto CometSlide. After gelling, lysis, electrophoresis, and DNA precipitation, the slides were stained with Green-DNA Dye (#163795–75–3) from Sangon Biotech and captured by fluorescence microscopy.

### IF assay

An IF assay was performed to detect the expression of ɣ-H2AX in GBM cells. Briefly, cells were collected, fixed, and opsonized. After blocking, the cells were incubated with an anti-ɣ-H2AX antibody (1:5000) at 4 °C overnight. Cells were then incubated with Alexa Fluor 594-labeled secondary antibody (1:2000). Hoechst 33342 (1:2000) was then used to stain the nuclei, and ɣ-H2AX-positive cells were captured under an Olympus FV1000 confocal fluorescence microscope.

### CHX chase assay

For this assay, 100 µg/ml CHX was added to cells transfected with the indicated plasmids. Cells were collected at the indicated time points, namely, 0 h, 1 h, 2 h, 4 h, and 8 h. The indicated proteins were detected by western blot analysis. The intensity of the protein was measured by a densitometer.

### IP assay

The IP assay was performed according to the manufacturer’s instructions. Briefly, protein A/G magnetic beads were preincubated with the different antibodies. Cells were collected and lysed with IP lysis buffer. Cell lysates were incubated with the antibody-coupled beads at 4 °C overnight. The beads were washed and then denatured at 100 °C, and the proteins were detected by western blotting.

### Ubiquitination assay

The ubiquitination assay was performed following a previous protocol.^43^

### Sumoylation assay

Endogenous sumoylated ATR was detected following a published protocol.^45,46^ In detail, cells were washed with cold wash buffer (10 mM NEM in PBS) and then lysed with lysis buffer (20 mM sodium phosphate pH 7.4, 150 mM NaCl, 1% SDS, 1% Triton, 0.5% sodium deoxycholate, 5 mM EDTA, 5 mM EGTA, 10 mM N-ethylmaleimide (NEM), and protease inhibitor and phosphatase inhibitor cocktails). Cell lysates were sonicated until they became fluid and then diluted 1:10 with dilution buffer (20 mM sodium phosphate, pH 7.4, 150 mM NaCl, 1% Triton, 0.5% sodium deoxycholate, 5 mM EDTA, 5 mM EGTA, 20 mM NEM, and protease inhibitor and phosphatase inhibitor cocktails). Lysates were then incubated with anti-ATR antibody coupled to protein A/G magnetic beads at 4 °C overnight. Beads were boiled in SDS sample buffer (125 mM Tris, pH 6.8, 5% SDS, 0.2% bromophenol blue, and 25% glycerol). Sumoylated ATR was detected by western blot using anti-SUMO2/3 antibody.

### Drug treatment

To detect the chemotherapeutic sensitivity of the indicated cells, cells infected with the indicated plasmids for 2 days were treated with the indicated concentration of TMZ for 3 days and DOX for 2 days.

### Soft agar assay

Colony-formation ability was examined with soft agar assays as described previously.^43^

### Xenograft assay

Four-week-old female nude mice were purchased and housed in an SPF room. A total of 1 × 10^5^ of the indicated cells were collected and resuspended in 10 µL PBS and then injected into the brains as described previously.^44^ When all mice injected with cells of the control group died due to tumor progression, the brains of all mice were collected, and stained with hematoxylin and eosin (H&E).

### Statistical analysis

Microsoft Excel was used for statistical analysis. FlowJo was used to analyze the cell cycle and apoptosis. Quantitative data are presented as the mean ± SEM, and analyzed using unpaired two-tailed *t* tests. Significant differences were computed by GraphPad and R software. *P*-values of < 0.05 (*) and < 0.01 (**) were considered statistically significant.

## Supplementary information

supplementary materials

## Data Availability

The data used to support the findings of this study are included within the article.
