# Supplementary material for: NUSAP1 potentiates chemoresistance in glioblastoma through its SAP domain to stabilize ATR
Source: Signal Transduct Target Ther. 2020 Apr 22;5:44. doi: 10.1038/s41392-020-0137-7 (PMC7174393; doi:10.1038/s41392-020-0137-7)
Supplement: Supplementary file 1 — supplementary materials [file 41392_2020_137_MOESM1_ESM.pdf]

Supplementary Materials for

NUSAP1 potentiates chemoresistance in glioblastoma through its SAP domain to stabilize ATR

Yuzu Zhao[1, 2], * Jiang He[1, 2], * Yongsen Li[1, 2], Shengqing Lv[3], # Hongjuan Cui[1, 2 #]

Correspondence to: hcui@swu.edu.cn or hongjuan.cui@gmail.com

This PDF file includes:

Figures. S1 to S5

Supplementary Fig. S1

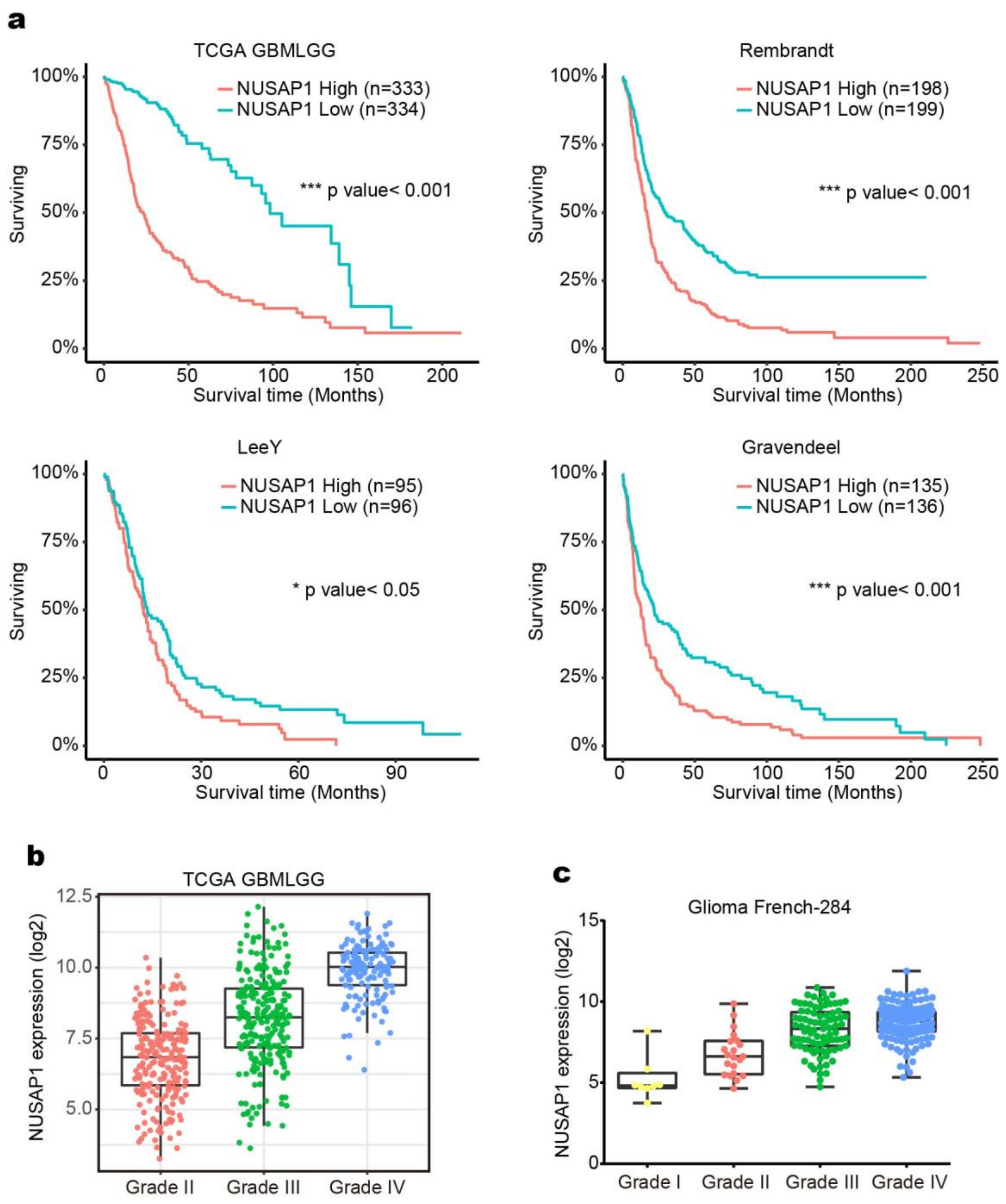

Figure. S1.

High level of NUSAP1 predicts poor prognosis. (a) Kaplan–Meier analysis of progression-free survival using data from 4 different glioma databases. (b) The level of NUSAP1 in grade (II–IV)

gliomas from TCGA GBMLGG database. (c) The expression of NUSAP1 in grade (I–IV) gliomas from French 284 database.

Supplementary Fig. S2

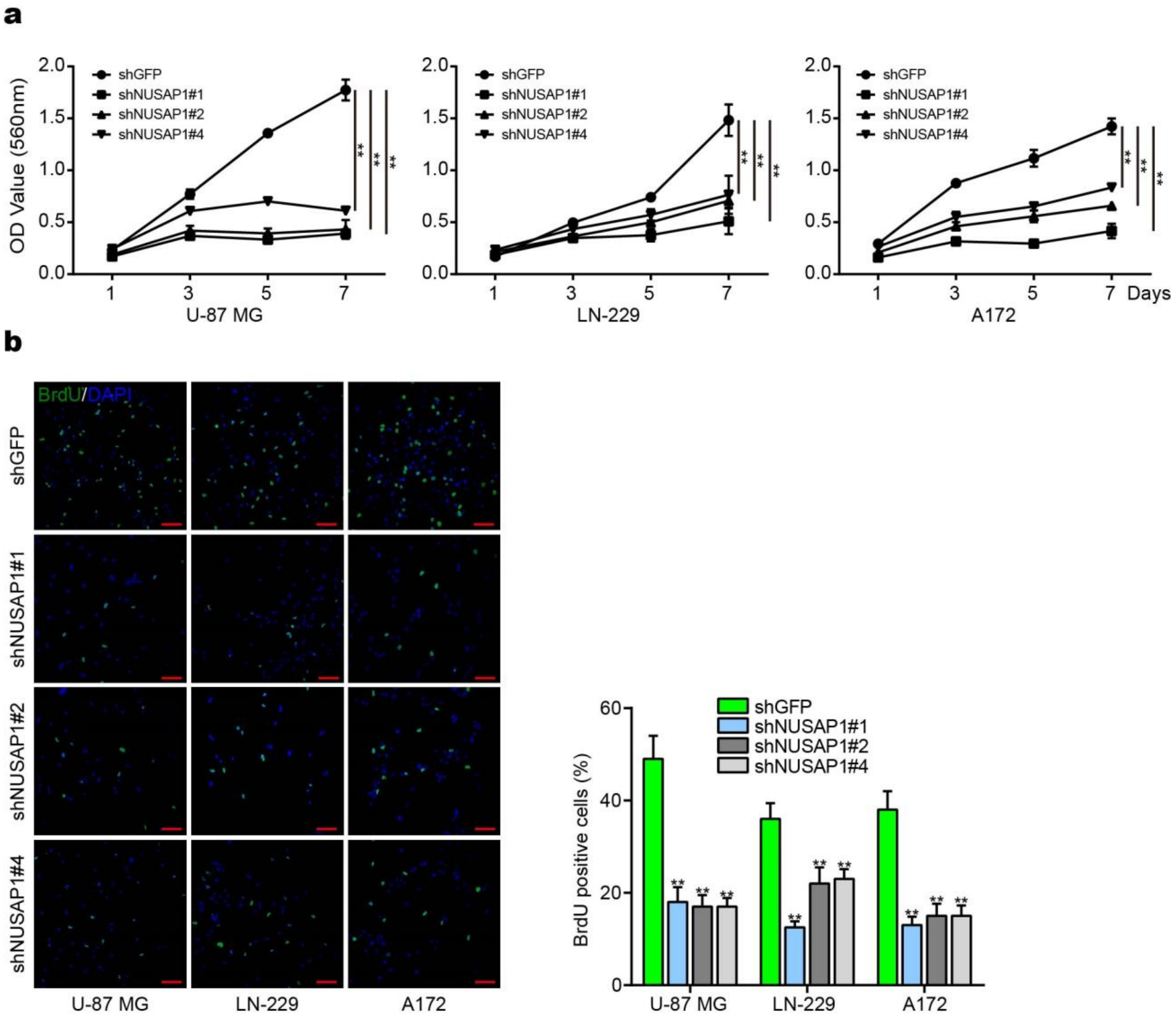

Figure. S2.

Knocking down of NUSAP1 inhibits cell viability and DNA synthesis. (a) Viability of NUSAP1-knockdown GBM cells. (b) BrdU-positive GBM cells after knocking down of NUSAP1.

Supplementary Fig. S3

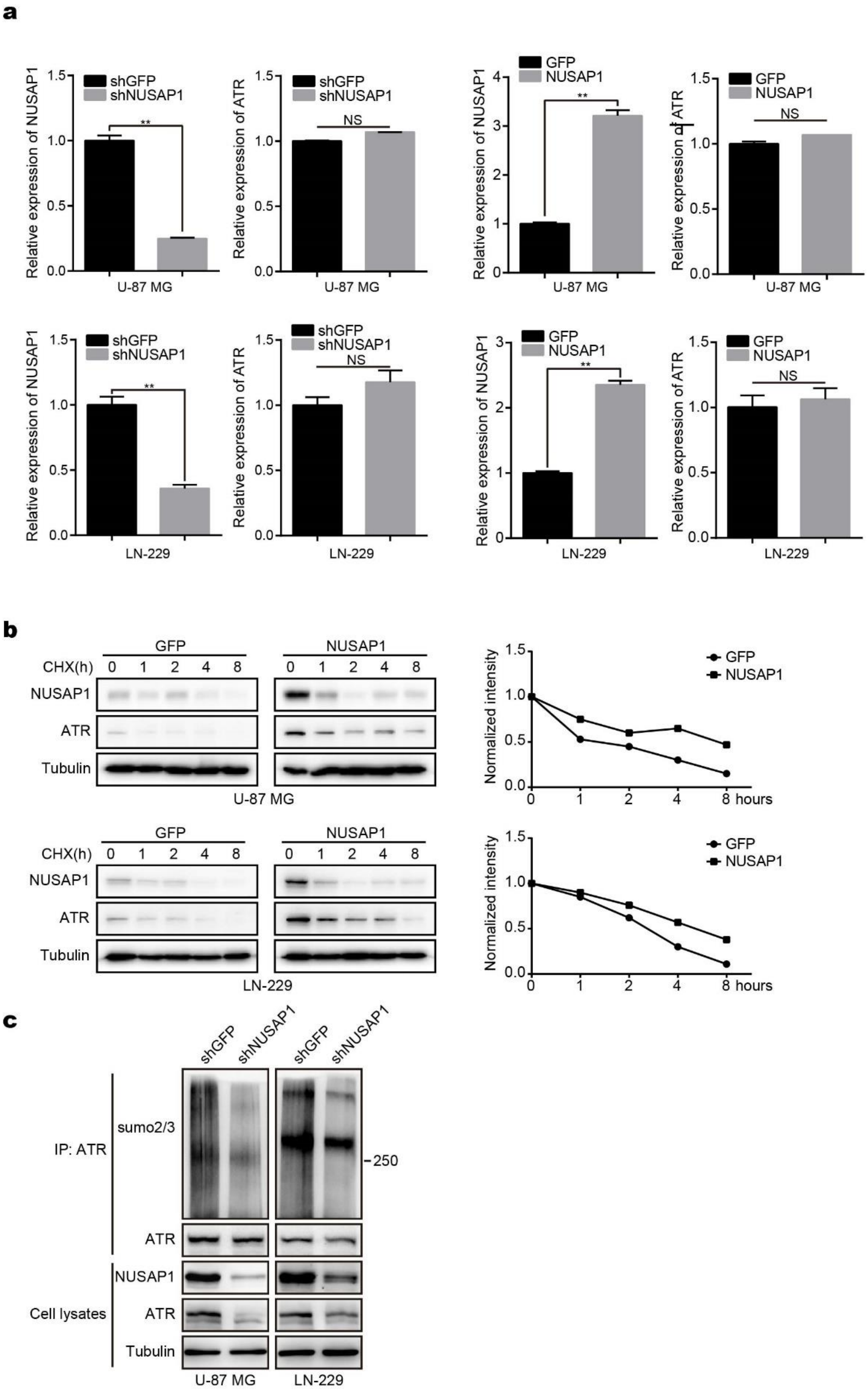

Figure. S3.

NUSAP1 stabilizes ATR in GBM cells. (a) mRNA level of NUSAP1 and ATR in indicated cells. (b) The level of indicated proteins in NUSAP1-overexpressed GBM cells treated with CHX for 0 h, 1 h, 2 h, 4 h, or 8 h. (c) The expression of sumoylated ATR in GBM cells knocking down of NUSAP1.

Supplementary Fig. S4

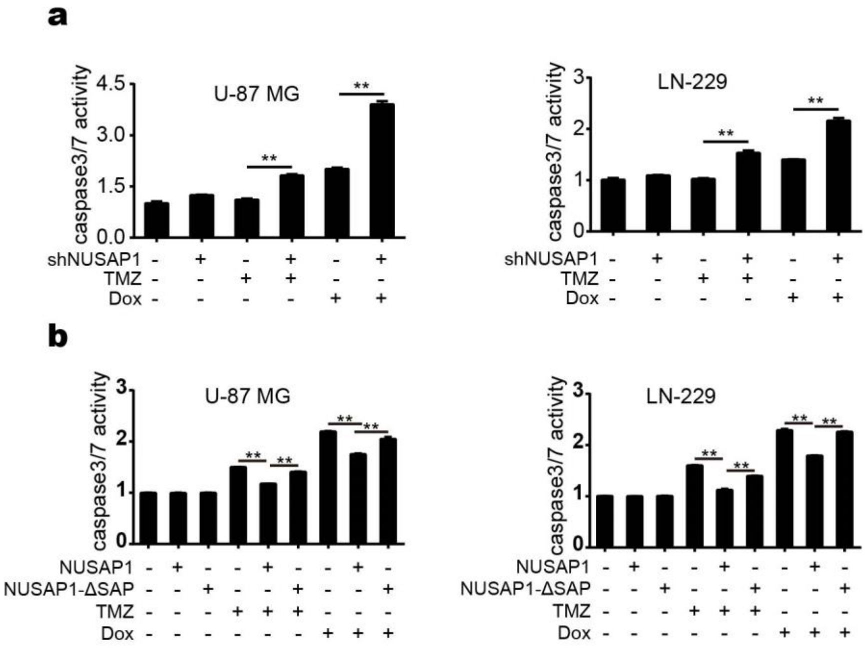

Figure. S4.

NUSAP1 contributes to chemoresistance of GBM cells. (a) Caspase3/7 activity of GBM cells depleting of NUSAP1 in response to TMZ and DOX respectively. (b) Caspase3/7 activity of NUSAP1 as well as NUSAP1-Δ SAP overexpressed GBM cells treated with TMZ and DOX respectively.

Supplementary Fig. S5

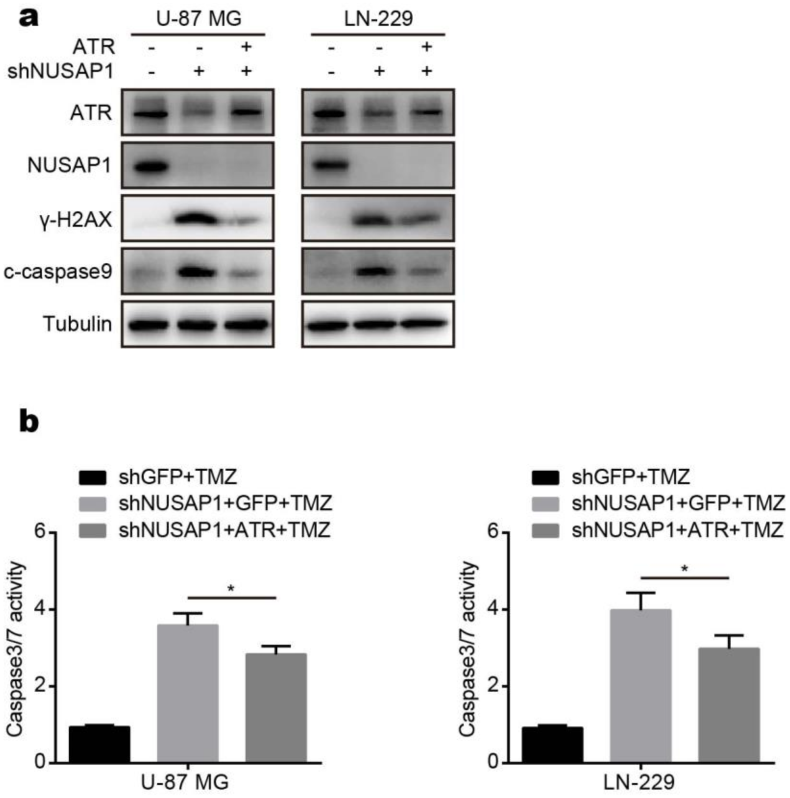

Figure. S5.

NUSAP1 participates in apoptosis, DNA damage and chemoresistance through ATR. (a) The level of γ-H2AX and cleaved-caspase9 in GBM cells overexpressed ATR after knocking down of NUSAP1. (b) Caspase3/7 activity of GBM cells overexpressed ATR after depleting of NUSAP1 in response to TMZ.
